# Deconstructing transcriptional variations and their effects on immunomodulatory function among human mesenchymal stromal cells

**DOI:** 10.1186/s13287-020-02121-8

**Published:** 2021-01-09

**Authors:** Changbin Sun, Kehua Zhang, Jianhui Yue, Shufang Meng, Xi Zhang

**Affiliations:** 1 BGI-Shenzhen, Jinsha Road, Dapeng New District, Shenzhen, 518083 China; 2 BGI Education Center, University of Chinese Academy of Sciences, Shenzhen, 518083 China; 3 China National GeneBank, BGI-Shenzhen, Shenzhen, 518120 China; 4 Cell Collection and Research Center, National Institutes for Food and Drug Control, Beijing, 100050 China; 5 Section of Cell Biology and Physiology, Department of Biology, University of Copenhagen, Copenhagen, Denmark

**Keywords:** MSC, RNA-seq, Expression variability, Immune licensing, Immunomodulatory potency

## Abstract

**Background:**

Mesenchymal stromal cell (MSC)-based therapies are being actively investigated in various inflammatory disorders. However, functional variability among MSCs cultured in vitro will lead to distinct therapeutic efficacies. Until now, the mechanisms behind immunomodulatory functional variability in MSCs are still unclear.

**Methods:**

We systemically investigated transcriptomic variations among MSC samples derived from multiple tissues to reveal their effects on immunomodulatory functions of MSCs. We then analyzed transcriptomic changes of MSCs licensed with INFγ to identify potential molecular mechanisms that result in distinct MSC samples with different immunomodulatory potency.

**Results:**

MSCs were clustered into distinct groups showing different functional enrichment according to transcriptomic patterns. Differential expression analysis indicated that different groups of MSCs deploy common regulation networks in response to inflammatory stimulation, while expression variation of genes in the networks could lead to different immunosuppressive capability. These different responsive genes also showed high expression variability among unlicensed MSC samples. Finally, a gene panel was derived from these different responsive genes and was able to regroup unlicensed MSCs with different immunosuppressive potencies.

**Conclusion:**

This study revealed genes with expression variation that contribute to immunomodulatory functional variability of MSCs and provided us a strategy to identify candidate markers for functional variability assessment of MSCs.

**Supplementary Information:**

The online version contains supplementary material available at 10.1186/s13287-020-02121-8.

## Background

Mesenchymal stromal cells (MSCs), also well-known as mesenchymal stem cells, are one of the most comprehensively studied multipotent cells with highly promising applications in immune modulation and regenerative medicine. To date, there are more than 1000 clinical trials registered in ClinicalTrials.gov (http://www.clinicaltrials.gov) attempting to utilize MSCs for cellular therapy [1, 2]. However, the clinical outcomes vary significantly among different cellular therapy trials. For example, in clinical usage of MSCs for treating therapy-refractory graft-versus-host disease (GvHD), only a small proportion of patients had favorable outcomes while many others did not show any apparent efficacy [3]. Functional variation and heterogeneity of MSCs, plus the lack of efficient assays for assessing MSC potency, could be some of the leading reasons resulting in inconsistent clinical outcomes [4].

MSCs have been isolated from various tissues, such as bone marrow [5], adipose tissue [6], umbilical cord [7, 8], and placenta [9, 10]. These cells comply with the minimal criteria defined by International Society for Cellular Therapy (ISCT) in 2006 [11] based on their morphological, phenotypic, and functional characteristics. However, recently, increasing number of MSC-based studies demonstrated that MSCs derived from different donors, tissues, and even sub-clones from the same cell line differed in their functional properties, such as immunomodulatory function, which will impact their applications [12–15]. Besides cell origins, the heterogeneity of MSCs could also be introduced by different isolation methods, culture media and methods, passage numbers, and/or freezing processes and lead to changes in proliferation and differentiation capacities, as well as in immunosuppression potency [16–18].

The unique immunomodulatory plasticity of MSCs makes them an invaluable cell type. MSCs exert their therapeutic effects through forming a balanced inflammatory and regenerative microenvironment, and their immunomodulatory capabilities are not constitutive but rather are licensed by inflammatory cytokines, such as INFγ and TNFα [1]. Licensed MSCs could release various cytokines (such as TGFβ, IL-10, CCL2, IL-6, IL-7, CXCL9, and CXCL10) [19, 20], growth factors (such as HGF and LIF) [21, 22], immunosuppressive molecules (such as NO, PGE2, TSG6, HO1, and galectins) [23, 24], and/or MSC-derived exosomes [25–27] to modulate differentiation, maturation, and inflammatory state of immune cells, such as dendritic cells (DCs), macrophages, and monocytes, and promote the formation of regulatory T (Treg) cells and prevent the activation of effector T cells [24]. In addition, MSCs responding to inflammatory environment could also upregulate immunosuppressive molecule PD-L1 which inhibits T cell activation [28] and FASLG which induces T cell apoptosis [29] through cell-to-cell interaction. Recent studies greatly improved our understanding in the immunoregulatory mechanisms of MSCs. However, why different MSC samples differ in immunomodulatory potency before licensing remains unclear and needs to be further investigated [1].

Here, we comprehensively investigated transcriptomic variations among MSC samples derived from multiple tissues. According to the expression patterns, we categorized these samples into 7 groups exhibiting distinct functional enrichment. To identify potential molecular mechanisms that result in distinct MSCs with different immune modulatory potency, we analyzed transcriptome changes of MSCs licensed with INFγ. Differential expression analysis indicated that different groups of MSCs deployed common regulation networks in response to inflammatory stimulation while expression variation of genes in the networks could trigger their different immunosuppressive capability. We also found that these different responsive genes showed high expression variability among unlicensed MSC samples. Finally, a gene panel was derived from these different responsive genes and was able to regroup unlicensed MSCs with different immunosuppressive potency.

## Methods

### Cell isolation and culture

MSCs were obtained from consenting subjects according to the Institutional Review Board of BGI (BGI-IRB for short) approved guidelines. Human placentas (*n* = 4) and umbilical cords (*n* = 7) were collected from naturally delivered full-term newborns. Dental pulps were obtained from donated wisdom teeth (*n* = 4, age 15–30).

Placenta-derived MSCs (PL-MSC) were isolated from chorionic plate (CP) after removing the amniotic membrane from the fetal surface of the placenta. After washing with PBS, PL were mechanically dissociated into tissue explants of approximately 2 mm^2^, which were then seeded into T75 flasks and cultured in MSC medium at 37 °C with 5% CO_2_ in a humidified atmosphere. MSC medium composed of MEMα (Hyclone), 1× L-glutamine (Thermo Fisher Scientific), 10% FBS (Hyclone), and 1× Antibiotic-Antimycotic (Thermo Fisher Scientific).

Umbilical cord-derived MSCs (UC-MSCs) were isolated from Wharton’s jelly (WJ) within the umbilical cord after dissection and removal of the arteries, vein, and amniotic epithelium. Tissue explants were applied to isolate and culture UC-MSCs using the same method as PL after tissue dissection.

Human dental pulp-derived MSCs (DP-MSCs) were isolated from DP uncovered by means of bone forceps to fracture the dental crown in several parts, as described previously [30]. Then, the dental pulp was enzymatically treated with 1 mg/ml type I collagenase (Sigma) and 3 mg/ml type II dispase (Sigma) for 1 h and cultured in MSC medium at 37 °C with 5% CO_2_ in a humidified atmosphere.

When cell density reached about 80% confluence, cells were dissociated with TrypLE™ Select (ThermoFisher Scientific) incubated at 37 °C for 2 min. The collected cells were passaged about every 3–5 days at a seeding density of 5000 cells/cm^2^. All assays were performed using MSCs between passages 2 and 5.

### IFNγ treatment and cell collection

MSCs were seeded into 6-well plates at a density of 5000 cells/cm^2^ and then cultured in MSC medium at 37 °C with 5% CO_2_ in a humidified atmosphere. When cell density reached about 70% confluence, MSCs were stimulated with IFNγ (5 ng/mL, R&D) for 24 h; meantime, parallel untreated wells were used as paired control. After treated for 24 h, the cells were lysed by adding of 1 ml TRIzol (Invitrogen) into each well after removing the medium and washed 3 times with PBS. For total mRNA extraction, each sample was pooled from 2 wells of 6-well plates cultured at the same time.

### RNA-Seq library construction and sequencing

Total mRNA was extracted using TRIzol (Invitrogen) reagent, as described previously [31]. Briefly, cells lysed by TRIzol were centrifuged and chloroform was added to the supernatant and mixed well. After spin, supernatant was mixed with chloroform/isopropanol (24:1) and centrifuged again. The same volume of isopropanol was added to the supernatant and stored at − 20 °C for 1 h, and then samples were centrifuged to precipitate RNA. RNA was washed by 75% alcohol twice and dissolved in nuclease-free water. The purity, integrity, and density of RNA were detected by Nanodrop, Agarose gel electrophoresis, and Agilent 2100 BioanaLyzer respectively, and then cDNA was synthesized and PCR was used to construct RNA-Seq library. All protocols for BGISEQ-500 library construction, preparation, sequencing, and quality control were provided by BGI.

To enhance the repeatability of our experiments, 13 cell lines (UC (*n* = 7), PL (*n* = 3), AD (*n* = 2), and PD (*n* = 1)) banked in the National Institutes for Food and Drug Control were independently cultured and treated with IFNγ with the similar methods as mentioned above. Then, the cells were lysed by TRIzol and shipped to our labs for RNA-seq.

### RNA-Seq data processing and quality control

To get public available RNA-seq data of MSCs, we searched in Gene Expression Omnibus using keywords “(MSC OR Mesenchymal stem cell OR Mesenchymal stromal cell) AND “Homo sapiens”[porgn:__txid9606]” with “Expression profiling by high throughput sequencing” type for data information collection. Then, we manually removed samples cultured with certain treatment, from donors with certain disease, with gene modification, or differentiated from ESCs, iPSCs, or other cell types (Fig. S1). We used Illumina platforms for sequencing and retained samples with reads not less than 10,000,000. Totally, we obtained 120 samples, of which raw files were downloaded from NCBI SRA database [32].

Quality control for each sample was performed by FastQC; adaptors and poor quality bases at read ends were trimmed by cutadapt [33] before mapping. Reads were mapped to the human genome (GRCh38) using HISAT2 with default parameters [34]. Raw counts of sequencing reads for the feature of genes were extracted by featureCounts [35]. MSCs RNA-seq data sequenced in our lab was also processed by the same mapping and feature counts extraction methods processes as mentioned above.

After read mapping and raw count extraction, we further compared the percentage of aligned exactly 1 time and median pairwise correlation coefficient for each sample, and we considered samples with percentage of aligned exactly 1 time that are lower than 60% and samples with median pairwise correlation coefficient *r* less than 0.9 as outliers (Fig. S2a) and left out for the further analysis. Finally, 69 downloaded samples together with our 20 untreated samples were used to investigate transcriptomic variation among MSC samples (Table S1).

### Filtering and normalization

Expressed genes in MSCs were defined as genes with counts per million (CPM) value more than 1 in at least 10% of the total samples; others were considered as none (not detected in all samples) or lowly expressed genes (CPM < 1 in at least 90% of the total samples), which were filtered out before normalization. The trimmed mean of *M* values (TMM) normalization method was used to estimate scale factors between samples and normalize RNA composition by calcNormFactors function in R package edgeR [36, 37].

### Variable-expressed gene identification

To quantify variability of gene expression across MSCs, distance-to-median (DM) statistic was used as a corrected version of coefficients of variation (CV), which is independent of the mean expression and gene length, as previously described [38]. Briefly, counts per million (CPM) were computed for the mean-corrected residual of the squared CV of each gene to its corresponding rolling median calculation. To correct for the effect of gene length on the mean corrected residual, DM was defined as the difference between the mean corrected residual of the squared CV of each gene and its expected residual from gene length. We computed the rolling median in 50 windows and set the number of overlapping genes between adjacent windows to 25 [38].

### Data dimension reduction, visualization, and clustering

R function *prcomp* with default parameters was used to perform principal component analysis (PCA) for expression of selected gene sets. R function *Rtsne* from Rtsne package was applied for visualizing high-dimensional data into a two-dimensional map in Fig. 3a, b with initial_dims = 10, and before running, we set seed to 1. A graph-based clustering approach [39] was used to cluster the samples into different groups. The first 10 PCs in the data were applied to construct an SNN matrix using the *FindNeighbors* function in Seurat v3 with k.param set to 10. We then identified clusters using the *FindClusters* command with the resolution parameter set to 2.

### Differential expression analysis

To identify differentially expressed genes (DEGs), we used R package edgeR to organize, filter, and normalize the data, and quasi-likelihood *F* tests were applied to identify DEGs according to the guide [37, 40]. Genes that differed in expression by two folds and with a false discovery rate (FDR) < 0.1 were assigned as DEGs.

### GO and pathway enrichment analysis

To find the GO and KEGG terms enriched in defined gene sets, we used the DAVID web-tool [41]. For figures, we only reported the top-ranked terms illustrated in the legends.

### Gene set enrichment analysis

Gene set enrichment analysis (GSEA) [42] was performed with 1000 permutations by GSEA_4.0.2 desktop application. Gene lists were ranked by the significant score defined as −log10(FDR) multiplied by log-transformed fold-change between two conditions or DM value for Fig. 2a, g, S3c, and S3d. Gene sets from the Molecular Signature Database (MSigDb) were used for GO and KEGG analysis. Gene sets that contained between 15 and 300 genes were included to provide more biologically meaningful results and reduce false positives.

### Pathway enrichment analysis and visualization

Pathway enrichment analysis was achieved according to protocol described in [43]. We downloaded the pathway gene set database Human_GOBP_AllPathways_ no_GO_iea_October_01_2019_symbol.gmt from the Bader lab dated October 01, 2019, for all pathway enrichment analyses. Gene lists were ranked by the significant score defined as −log10(FDR) multiplied by log-transformed fold-change between two conditions. After gene set enrichment analysis (GSEA), pathway sets that contained between 15 and 300 genes were included to provide more biologically meaningful results and reduce false positives.

For map visualization, pathway enrichment analysis results were interpreted in Cytoscape using its EnrichmentMap, AutoAnnotate, WordCloud, and clusterMaker2 applications [43, 44]. The pathway enrichment map was created with parameters FDR *Q* value < 0.001 and combined coefficient > 0.375 with combined constant = 0.5 [44].

### MSC and PBMC co-culture for immunosuppressive potency assessment

The suppressive effect of MSCs on leukocyte expansion was confirmed as described previously [45]. Briefly, MSCs were seeded into 24-well plates at a density of 1 × 10^5^ cells per well, and CFSE (Sigma)-labeled human PBMCs were added to each MSCs well at a 1:5 (cell number) co-culture ratio of MSCs to PBMCs. Then, 10 μg/mL phytohemagglutinin (PHA) (Sigma) was used to activate PBMC cells. PBMCs at a same density without MSCs and PHA were used as negative control. For positive control, we plated the same number of PBMCs/well with PHA to active leukocytes. After 5 days of co-culture, PBMCs were collected and measured using a FACSCalibur platform (BD Biosciences). The suppression of T cell proliferation by MSCs was calculated as [100% − (T cell proliferation after co-culture with MSCs divided by positive control × 100)%]. Negative control was applied to define a threshold of the CFSE signal of non-proliferating T cells.

## Results

### Data selection and quality control

**Fig. 1:**
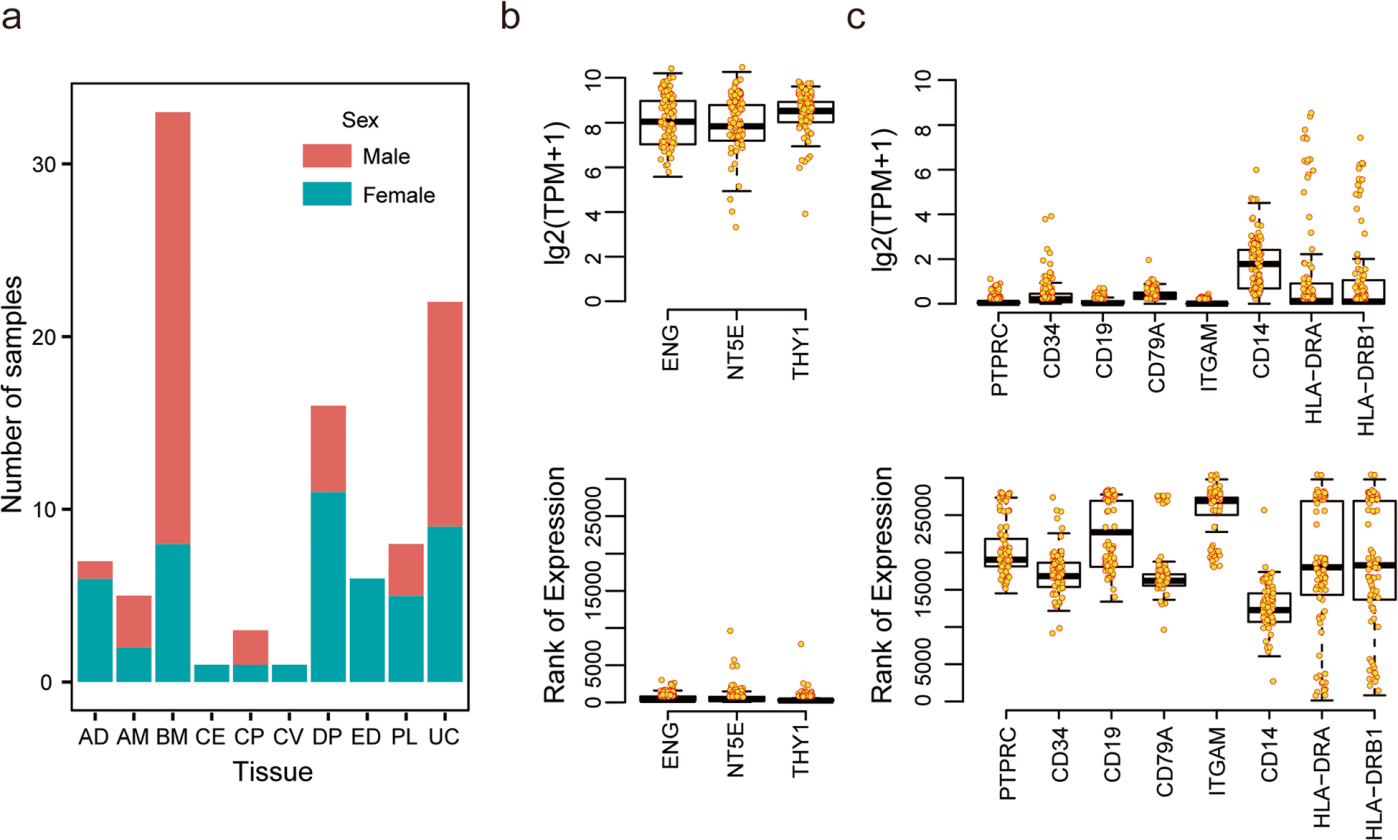
Overview of data collected for transcriptome variation analysis. **a** Barplot showing number of samples derived from different tissue origins used for transcriptome variation analysis. **b**, **c** Boxplots showing distribution of expression (up) and ranking (down) for positive markers (**b**) and negative markers (**c**) across samples, respectively. Some MSC samples, including AM CM, CP, and CV, derived from different anatomical parts of placenta. AD, adipose tissue; AM, amniotic membrane; BM, bone marrow; CM, chorionic membrane; CP, chorionic plate; CV, chorionic villi; ED, endometrial; DP, dental pulp; PL, placenta; UC, umbilical cord

To comprehensively investigate transcriptomic variations among MSC cell lines cultured in vitro, RNA-seq data of total 102 samples were integrated for gene expression analysis, of which 69 samples were selected from public database and 33 samples were newly sequenced in this study (Fig. S1 and S2; Table S1). Overall, the MSC samples were derived from 6 tissues (Fig. 1a) in 17 studies (Fig. S2b), including adipose tissue (AD), bone marrow (BM), dental pulp (DP), endometrial (ED), placenta (PL), and umbilical cord (UC). Of these samples, number of reads were mostly between 10,000,000 and 60,000,000, and more than 60% aligned exactly 1 time to the transcriptome (Fig. S2c), indicating high quality of these collected RNA-seq data.

Minimal criteria of defining MSC claimed that MSCs must express three positive markers, i.e., CD105 (ENG), CD73 (NT5E), and CD90 (THY1), and lack expression of several negative markers, including CD45 (PTPRC), CD34, CD14 or CD11b (ITGAM), CD79a (CD79A) or CD19, and HLA-DR (HLA-DRA and HLA-DRB1 etc.) [11]. Indeed, gene expression level ranked by TPM (transcripts per kilobase million) showed that those positive markers were highly expressed (Fig. 1b) while negative markers were weakly or not expressed in our samples, except HLA-DR molecules showed highly variable expression across samples (Fig. 1c). Considering that MSCs express HLA-DR surface molecules not only in response to stimulations, such as IFNγ, but also under some normal expansion culture conditions [46, 47], therefore, we did not remove these samples with higher expression of HLA-DR for the following gene expression analysis.

### Expression variations and characteristics among MSC samples

**Fig. 2:**
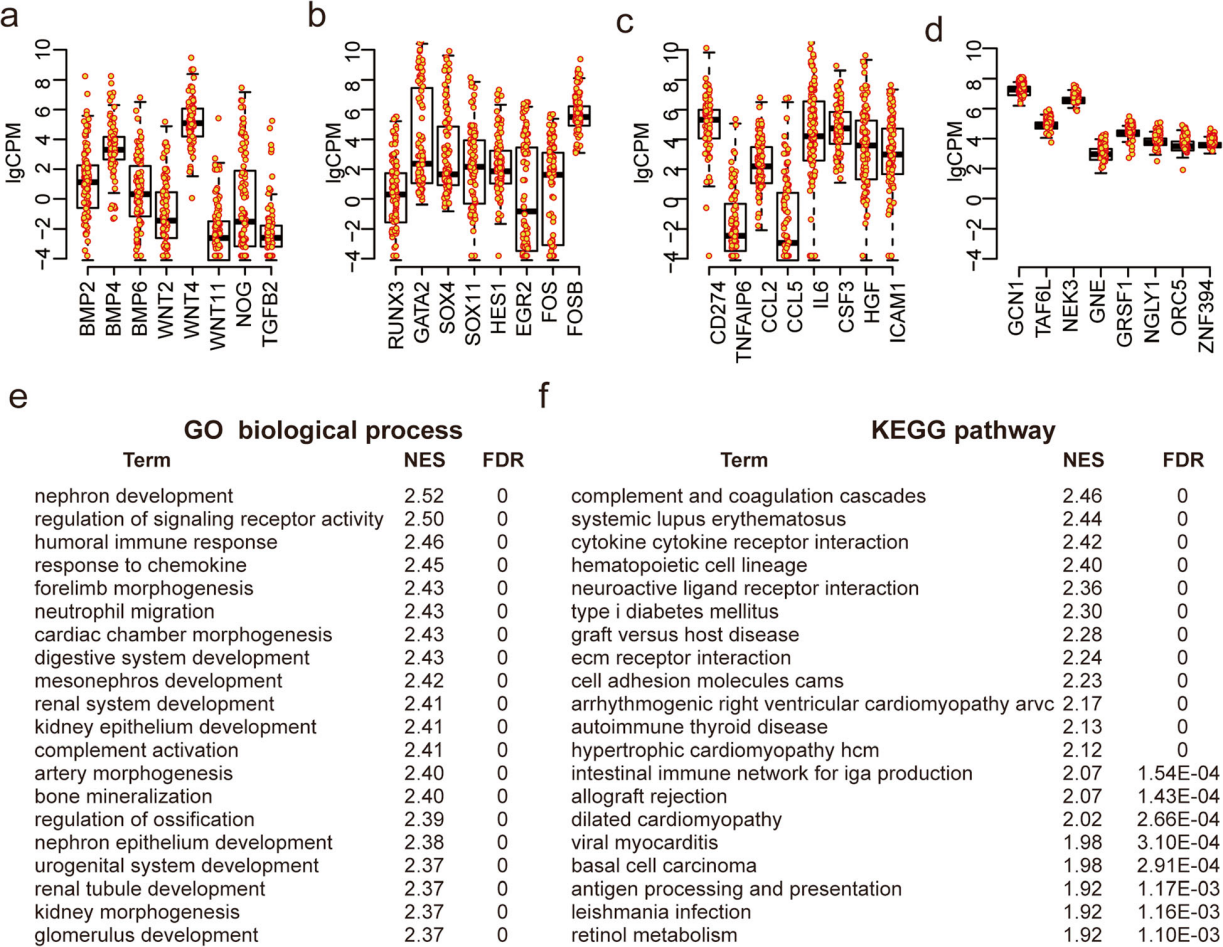
Transcriptomic variations across MSC samples. **a**–**d** Expression distribution of certain signaling-pathway related genes (**a**), transcription factors (**b**), and immune-related genes (**c**) with higher DM value compared to metabolic process-related genes (**d**). **e**, **f** GESA positive results showing enrichment in GO biological process (**e**) and KEGG pathway (**f**) gene sets database based on ranked genes list in descending order by the DM value. Only the top 20 terms with highest NES were presented (*p* < 0.001)

To analyze transcriptomic variations across MSC samples, the DM value was used to rank variations of genes expressed in MSCs. A larger DM value indicated a greater variation (Fig. S3a and S3b; Table S2). Interestingly, we noticed that growth signaling genes such as *BMP2/4/6*, *WNT2/4/11*, *NOG*, and *TGFB2*, as well as transcript factors such as *RUNX3*, *GATA2*, *SOX4*, *SOX11*, *HES1*, *EGR2*, *FOS*, and *FOSB*, which are involved in differentiation process of MSCs [48–54], were among the top genes with large DM values (Fig. 2a, b). Meanwhile, genes that regulated immunomodulation [1, 19, 55], such as *CD274* (PD-L1), *TNFAIP6* (TSG6), *CCL2*, *CCL5*, *IL6*, *CSF3*, *HGF*, and ICAM1, also exhibited highly variable expression among those samples (Fig. 2c). In contrast, genes involved in several other biological processes, such as metabolic process, gene expression, RNA process, and RNA binding, had very small gene expression changes (Fig. 2d). Therefore, we hypothesized that gene expression variations among MSCs may be used to select MSCs with different immunomodulation potency and differentiation propensity.

To further investigate whether expression variations among the MSC samples were related to specific MSC biological functional properties or not, we next performed gene set enrichment analysis (GSEA) with above pre-ranked gene list. Notably, genes with highly variable expression were significantly enriched in immune modulation and developmental process, such as humoral immune response, response to chemokine, nephron development, cardiac chamber morphogenesis, and digestive system development (Fig. 2e, f). Expression variability was also overrepresented in coding proteins located in extracellular matrix and cell surface, involving in cytokine, receptor regulator, and binding activity (Fig. S3c). On the other hand, genes with stable expression were housekeeping genes involved in basic cellular function, such as RNA processing (Fig. S3d). These results together demonstrated that transcriptome-wide sample-to-sample variations among MSCs were associated with their functional properties.

### Grouping MSC samples based on highly variable genes

**Fig. 3:**
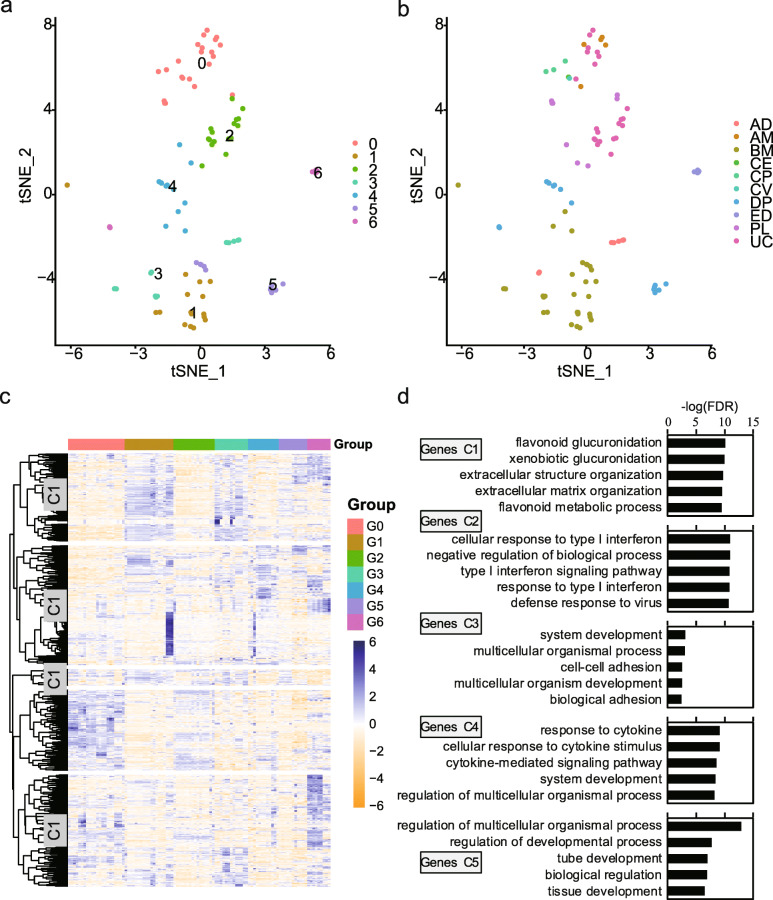
Groups and functional enrichment in MSCs. **a**, **b** tSNE visualizing the results of samples clustering (**a**) and tissue origins (**b**). **c** Heatmap showing expressive characteristics of HVGs among the groups. **d** Results of GO biological process enrichment analysis for each genes cluster. Top 5 terms with lowest *p*-adjusted value were presented

To identify candidate groups of the MSC samples based on gene expression pattern, genes with DM value more than 1 as highly variable genes (HVGs) were selected for data dimension reduction and clustering. The results showed that the samples we collected could be clustered into 7 groups (G0–G6) (Fig. 3a). Among these groups, G1 included samples mostly derived from BM while G3 included all AD-MSCs plus some BM-MSCs (Fig. 3b; Table S3). Other groups included MSC samples derived from different origins as well (Fig. 3b; Table S3). Meanwhile, the HVGs we selected grouped into five clusters with distinct functional enrichment, such as system development, tube development, metabolic process, and response to cytokine (Fig. 3b), indicating that different groups of MSC samples may have different differentiation propensity and immunomodulatory potency. In addition, different expression analysis among these groups demonstrated that functional enrichment of DEGs among these groups are associated with MSC function-related properties as well, such as angiogenesis, nervous system development, cell migration, and inflammatory response. Taken together, these results demonstrated that MSCs from different tissue origins could be classified into the same group with similar functional enrichment based on expression of HVGs, although tissue origins have been reported to impact greatly on functions of MSCs [56, 57].

To illustrate expression difference in these groups, we presented results obtained from comparison among G0 (*n* = 22, 15 downloaded, 7 newly sequenced), G2 (*n* = 15, 1 downloaded, 15 newly sequenced), G3 (*n* = 13, 11 downloaded, 2 newly sequenced), and G4 groups (*n* = 12, 3 downloaded, 9 newly sequenced) (Table S4), to which our 33 MSC samples were assigned (Table S3). GO biological process enrichment analysis demonstrated that G0 significantly upregulated genes involved in response to stimulus and inflammatory response, including cytokines such as *CXCL2*, *CXCL3*, *CXCL5*, and *CXCL20* (Fig. S4A and B). The upregulated genes in G2, G3, and G4 were overrepresented in developmental process with distinct developmental cell types. For example, genes related to nervous system development, circulatory system development, and nephron development were respectively upregulated in the G2, G3, and G4 groups (Fig. S4C-S4H). Altogether, we found significant gene expression variations existing among MSCs that could potentially influence their functional properties. Therefore, we hypothesized that quantitative RNA analysis of selected genes from HVGs could serve as a candidate matrix assay for characterizing MSC potency [58].

### Characteristics of expression changes in MSCs upon IFNγ licensing

Although above functional enrichment analysis demonstrated that some inflammatory response-related genes were over-represented in G0 (Fig. S4), it was not clear how these differences would affect MSC immunomodulatory behavior. Due to the critical role of IFNγ in licensing MSC-mediated T cell suppression [28], IFNγ, which can be used as an alternative for human PBMCs as responder cells in a MSC potency assay [19], was used to treat 27 MSCs within the G0 (*n* = 4), G2 (*n* = 15), and G4 (*n* = 8) groups to study response variation on transcriptomic level. For clarity, we assigned them as ssG0, ssG2, and ssG4 due to their small size compared to the number of samples in each group.

**Fig. 4:**
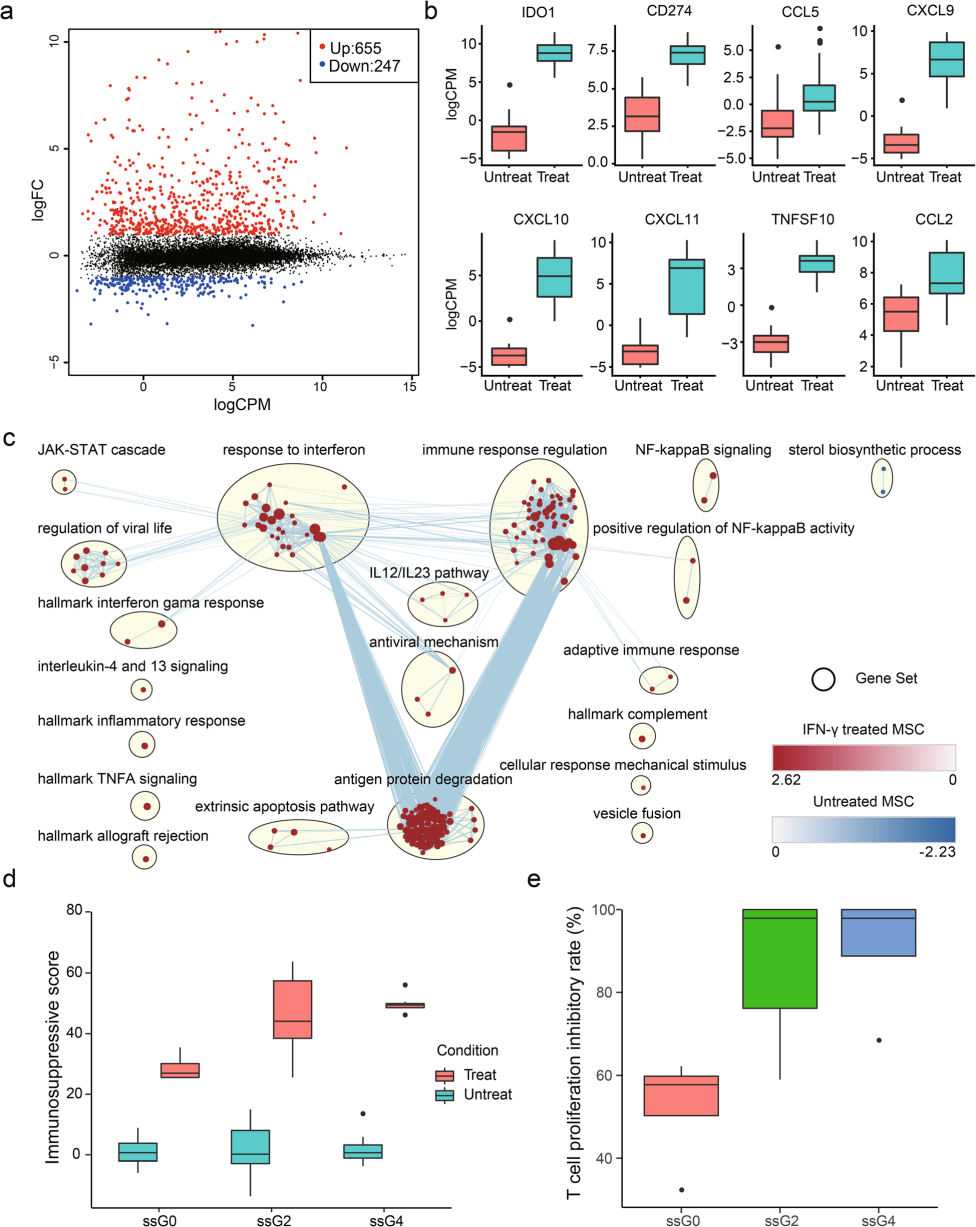
Gene expression changes in MSCs treated with IFNγ. **a** A mean difference plot showing DEGs identified in MSCs treated with IFNγ. **b** Representative genes upregulated in IFNγ-licensed MSCs. **c** Enrichment map showing pathways enriched in INFγ-treated MSCs (red) and untreated MSCs (blue). **d** Boxplot showing immunosuppressive scores for each group calculated based on VEGF, IFNa, CXCL10, GCSF, CXCL9, IL-7, and CCL2 expression level. **e** Results showing MSCs from ssG0, ssG2, and ssG4 co-cultured with PBMC for immunosuppressive potency assessment

Comparing to their paired untreated samples, we identified 902 genes that were differentially expressed (655: 72.62% upregulated vs 247: 31.26% downregulated) (Fig. 4a, Table S5). In line with previous studies, *IDO1*, the dominant determinant of MSC-mediated inhibition of T cell proliferation, and chemokines, including *CCL5*, *CXCL9*, *CXCL10*, and *CXCL11*, were upregulated in MSCs upon IFNγ licensing, which could potentially form a chemokine-IDO axis to exert immunoregulatory effects on various immune cells (Fig. 4b) [19, 59–61]. In addition, cytokines, including *CCL2*, *CCL7*, and *IL6*, apoptosis inducer *TNFSF10* (TRAIL), immune checkpoint proteins, including *CD274* (PD-L1) and *PDCD1LG2* (PD-L2), cell adhesion molecules, including *ICAM1* and *VCAM1*, and class II major histocompatibility complex (MHC), including *HLA-DRA*, *HLA-DRB1*, and *HLA-DRB5*, were also overexpressed in IFNγ activated MSCs (Fig. 4b and Table S5). Similarly, these genes have also been shown to play critical roles in MSC-mediated immunosuppression in previous studies [58]. Meanwhile, IFNγ licensing triggered specific signaling pathways in MSCs, such as upregulation of *JAK2*, *JAK3*, *STAT1*, *STAT2*, *SOSC1*, *SOSC3*, *TLR2*, and *TLR3*, to orchestrate their immune response. Pathway enrichment map further illustrated that IFNγ-licensed MSCs upregulated several gene clusters linked to response to interferon, immune response, and antigen and protein degradation (Fig. 4c). Interestingly, sterol biosynthetic process was downregulated in IFNγ-treated MSCs (Fig. 4c). Sterols are the major component of the cellular membranes and are essential for mammalian cell growth [62]. Decreased sterol synthesis could partially explain why IFNγ leads to a cytostatic response in MSCs [60]. Overall, a panel of immunomodulation-related genes was upregulated upon IFNγ licensing, and here we define these upregulated genes as common response genes (CRGs).

To predict T cell suppression potency, the sum of log normalized expression of VEGF, IFNa, CXCL10, GCSF, CXCL9, IL-7, and CCL2 genes, which were correlated with T cell suppression capacity of MSCs according to previous studies [19, 63], was calculated and served as MSC immunosuppressive score for the 27 MSCs within ssG0, ssG2, and ssG4 treated with IFNγ. Our results showed that MSC immunosuppressive score in ssG0 was significantly lower than those of ssG2 and ssG4 (Fig. 4d). MSC and PBMC co-culture experiments in vitro were performed on 16 MSCs (4 from ssG0, 8 from ssG2, and 4 from ssG4), and the results demonstrated that T cell proliferation inhibitory rate of G0 was significantly lower than those of ssG2 and ssG4. Taken together, these results demonstrated that different groups of MSCs clustered by their expression patterns of HVGs across unlicensed MSC samples could have distinct immunosuppressive capability, which may reflect on their different responses to inflammatory environment.

### Transcriptional variations of MSCs in response to inflammatory environment imitated by IFNγ treatment

**Fig. 5:**
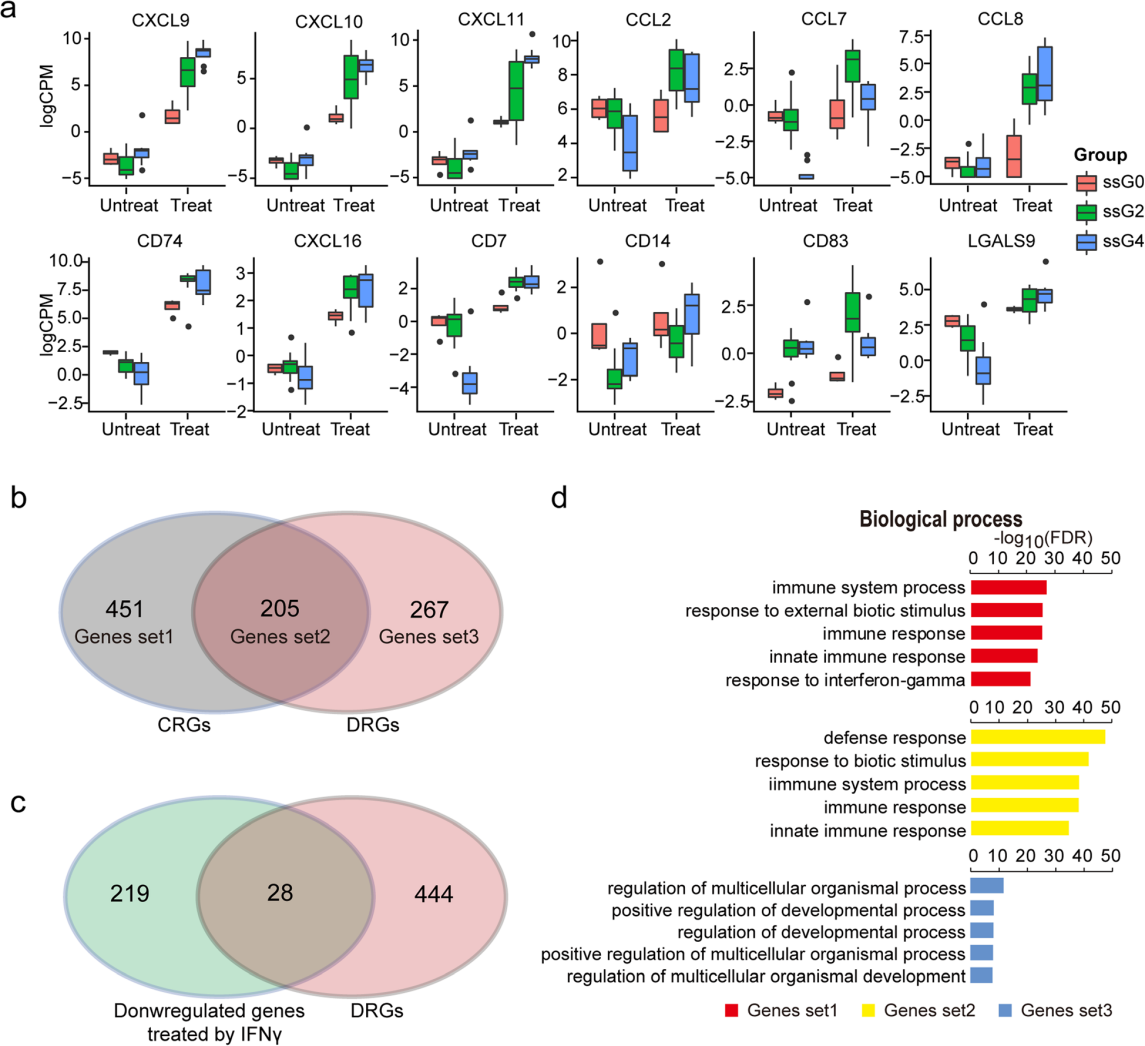
Expression variability of MSCs in response to IFNγ. **a** Representative genes showing different responsive to IFNγ licensing. **b** Venn diagram showing number of genes overlapped between CRGs and DRGs. Genes set1, Genes set2, and Genes set3 represented unique genes in CRGs, shared genes, and unique genes in DRGs respectively. **c** Venn diagram showing number of genes overlapped between genes downregulated in MSCs treated with IFNγ and DRGs. **d** Results of GO BP enrichment analysis for the Genes set1, Genes set2, and Genes set3

To identify genes that respond differentially to IFNγ licensing among the ssG0, ssG2, and ssG4 groups, we performed differentially expressed analysis and obtained a total of 472 genes defined as different response genes (DRGs). Most of the DRGs downregulated in ssG0 while upregulated in ssG2 and ssG4 are enriched in immune response pathways (Fig. S5A-S5D), including several well-known immune-modulating genes, such as *CXCL9*, *CXCL10*, *CXCL11*, *CCL2*, *CCL7*, *CCL8*, *CD74*, *CXCL16*, *CD7*, *CD14*, *CD83*, and *LGALS9* (Fig. 5a; Table S6). These genes are tightly involved in immunomodulatory processes, such as regulation of immune cell migration, T cell development and differentiation, T cell chemotaxis, immune activation, and cell survival [64–67].

To further identify the genes related to common immune-regulatory pathways and to differences in immunomodulatory capacities, we performed comparison between DRGs and CRGs. The genes unique to CRGs, shared between CRGs and DRGs, and unique to DGRs were designated as Genes set1, Genes set2, and Genes set3, respectively (Fig. 5b). Among the 472 DRGs, 205 genes were shared with CRGs and fall within Gene set2 (Fig. 5b and c; Table S7). Interestingly, functional enrichment analysis revealed that Genes set1 and Genes set2 were significantly involved in immunomodulatory functions, while Genes set3 was involved in regulation of developmental process (Fig. 5d), implying that IFNγ-treated MSCs may partly influence on their developmental behaviors. Taken together, these results suggested that MSCs exert immunosuppressive effects through common mechanisms, while variations in expression of some immunomodulatory genes upon inflammatory priming could result in their distinct immunosuppressive potency.

### Refining gene panel for grouping unlicensed MSCs with predictable immunosuppressive potency

According to our results, differential expression of certain genes under IFNγ-licensed state could potentially explain for differences in their immunomodulatory activity. However, there is still a lack of reports on genes whose expression in MSCs under unlicensed condition will be related to MSCs’ immunomodulatory potency.

**Fig. 6:**
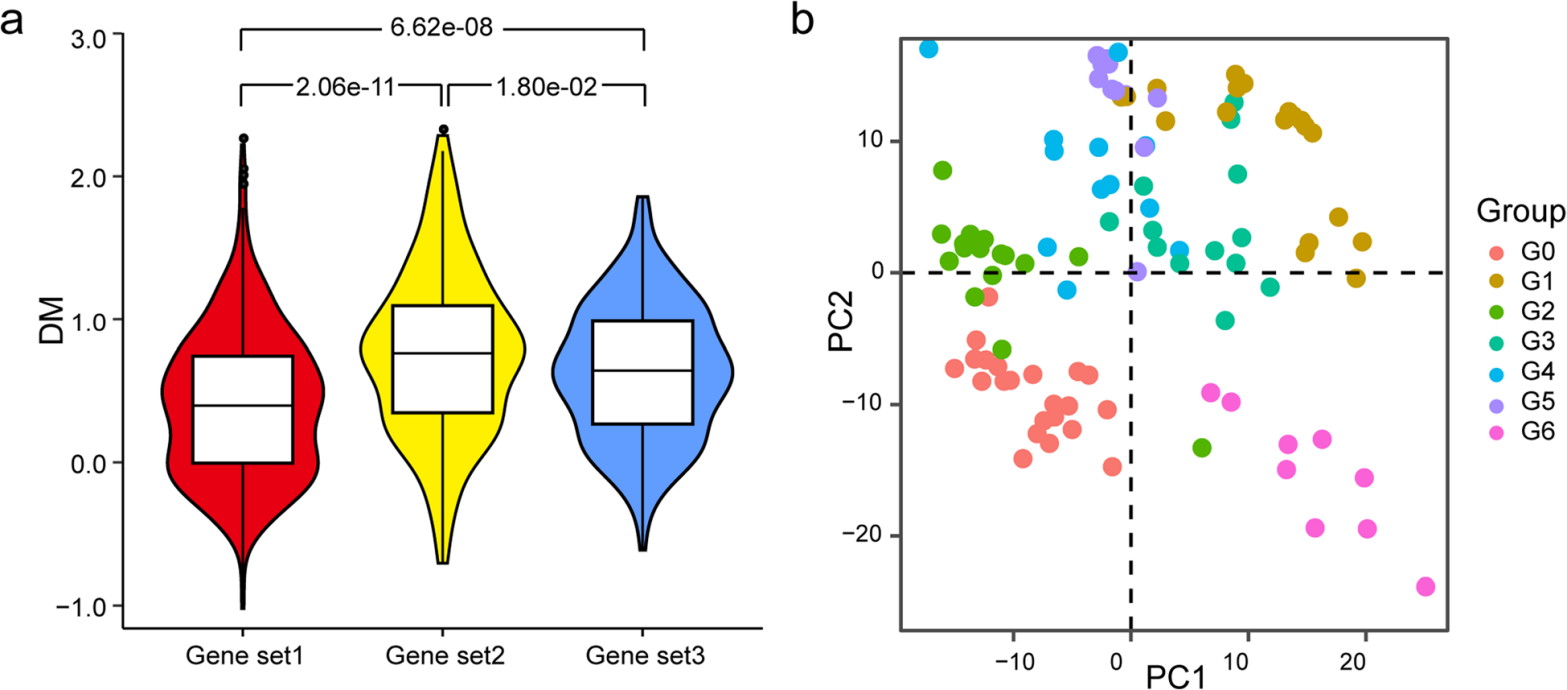
Candidate markers for MSC immunomodulatory potency assay. **a** Violin plot showing DM values distribution for the Genes set1, Genes set2, and Genes set3. *p* value were presented for one set compared to another performed by Wilcoxon rank sum test. **b** Scatter plot visualizing PCA results using top 100 genes with the highest DM values in the Genes set2 expressed in the untreated samples

Considering that genes in Genes set2 were not only related to common immune-regulatory pathways but also to differences in immunomodulatory capacities (Fig. 5b, c), differential expression of these genes under unlicensed condition may contribute to distinct immunomodulatory behavior of MSCs in response to inflammatory environment. Interestingly, when we compared DM distribution of genes in Genes set1, Genes set2, and Genes set3 in the total unlicensed MSC samples, genes in Genes set2 demonstrated significantly higher variation than Genes Set1 (*p* = 2.06e−11) and Genes Set3 (*p* = 1.80e−02) (Fig. 6a). Several immune response-related genes, such as *CCL2*, *CCL7*, *CD74*, *TNFSF10*, *LGALS9*, *IFIT1*, *VCAM1*, and *ICAM1*, fall within Genes set2 and were among the top highly variable genes (Fig. 2c and Table S2). These results indicated that expression variation of genes in Genes set2 may exert greater influence during immune activation of unlicensed MSCs.

Then, we applied the top 100 genes with highest DM values in Genes set2 as a gene panel and utilized their expression for data dimension reduction. The results of principal component analysis (PCA) showed that all MSC samples in the G0 group laid in the third quadrant while majority of the samples from the G1 to G5 groups laid in the first and second quadrant based on principal component 1 and principal component 2 (Fig. 6b). Since the G0 group exhibited the lowest immunosuppressive capacity compared to the G2 and G4 groups in both in silico and in vitro analysis (Fig. 5e), samples fall within the same quadrant as G0 may have lower immunosuppressive capability as well. Meanwhile, with INFγ-treated samples included, PCA analysis showed that the G6 group, of which all samples laid in the fourth quadrant, were closer to INFγ-treated samples (Fig. S6), indicating pre-licensed state of these samples. Taken together, our results demonstrated that the gene panel we selected here would be valuable for characterizing MSC immunomodulatory potency based on quantitative RNA-seq analysis.

## Discussion

Since the first report on the characteristics of MSCs derived from human BM [68], studies revealed that MSCs can be isolated from both pericytes and adventitial progenitor cells from nearly all tissues [69–71]. Although MSCs have been widely accepted as one of the most promising cell products to treat various degenerative and inflammatory disorders, such as graft versus host disease (GvHD), Crohn’s disease, multiple sclerosis, and diabetes, there are still clinical challenges, such as why the outcomes of advanced clinical trials were not as encouraging as pre-clinical animal data in a wide array of disease models [17]. In addition to limited understanding of mechanisms of action which MSCs deployed to regulate their anti-inflammatory and tissue repair functionalities, functional variability and heterogeneity could hinder development of effective assays for MSCs as potency release criterion for the advanced clinical trials [1, 17, 58]. These variability and heterogeneity manifest among donors, among tissue sources, as well as within cell populations [72–75]. Besides, distinct cell separation and preservation methods, culture media, and number of passages can affect cell functionality. For example, human umbilical cord blood mononuclear cells tested before and after cryopreservation showed different abilities to treat stroke [76], aged MSCs underwent morphological, phenotypic, and differentiation potential changes [77], and long-term culture increased genetic instability in MSCs [78]. These studies indicated that MSCs with distinct cell preparation, fitness, culture methods, and expansion levels could differ in their tissue-protective and immunomodulatory properties [17, 19]. However, the molecular contributors to the functional variability and heterogeneity remain unclear. Here, we analyzed RNA-seq from 102 MSC samples derived from 6 different tissues, including AD, BM, ED, PD, PL, and UC, to investigate their expression variations and relationship with their functional variability and identify candidate markers differently expressed influencing on their immunosuppressive potency.

Several studies have been done to compare gene expression similarity and variability among MSC samples [79–82]. These studies demonstrated that different gene expression profiles could reflect the ontogenetic sources of MSCs and indicate their distinct differentiation potential or other functional properties. In line with these studies, our results also showed that MSCs were mostly grouped together with the same tissue origin according to the expression pattern of HVGs (Fig. 3a, b; Table S3). However, these researches largely focused on expression difference among MSCs with distinct tissue origins while ignoring that MSCs from the same tissue might also exhibit functional variability. The functional differences could come from a variety of cues, including chemical, physical, and biological factors, expansion level, and characters of donors, which may result in changes of MSC functional characteristics [80, 83]. For example, compared to younger counterparts, aged MSCs from the same tissue with identical culture method display delayed clonogenic capacity and pro-inflammatory SASP-like phenotype, and their immunomodulatory properties were significantly reduced [83]. To address this, we performed data dimension reduction and clustering based on a nonparametric clustering technique (see the “Methods” section) to group these collected samples in the present study. Our results demonstrated that MSCs can be clustered into groups with diverse functional properties characterized by enrichment analysis (Fig. 3 and S4). Besides, MSCs from different tissues can be classified into the same group while MSCs from the same tissue as well can be clustered into different groups according to the expression patterns of HVGs (Fig. 3a, b; Table S3), indicating the importance of potency assays for MSCs before clinical trials or application.

Despite different tissue sourcing, our results are in line with that MSCs likely share fundamental mechanisms of action mediating their anti-inflammatory processes [58]. Our data are in agreement with reports that immunosuppression related molecules, such as *IDO1*, *CCL5*, *CXCL9*, *CXCL10*, *CXCL11*, *CD274*, *TNSF10*, *CCL2*, and *FLT3LG* (FLT3L), were upregulated upon INFγ licensing (Fig. 4b; Table S6) [60, 61], of which some were lowly or not expressed in unlicensed MSCs. Activated MSCs produce chemokines CCL5, CXCL9, CXCL10, and CXCL11, which could recruit T cells to the proximity of MSCs and suppress the proliferation and activity of T cells in their vicinity by expressing tryptophan catabolism rate-limiting enzyme IDO1 through metabolite kynurenic acid and/or by expression the immune checkpoint protein CD274 through cell-to-cell interaction [1, 84, 85]. In addition, a recent study demonstrated that MSCs might utilize IFNγ-FLT3L-FLT3 axis to suppress inflammation in lupus through upregulating tolerogenic DCs [86]. Different groups of MSCs should deploy shared regulation networks to exert immunosuppressive function upon IFNγ licensing, including JAT-STAT, NF-kappaB, IL-12/IL23, response to interferon, immune response, antigen and protein degradation, extrinsic apoptosis pathway, and complement system signaling pathways, which could form a regulatory network to orchestrate MSC immunomodulatory function (Fig. 4c). However, gene expression variations could result in different responses among MSC groups treated with INFγ, and these immunomodulation related genes were mainly in the shared regulation networks, including abovementioned *TNSF10*, *CXCL9*, *CXCL10*, *CXCL11*, and *CCL2*. Furthermore, human MSCs licensed by INFγ have been tested in NOD-SCID mice showing enhanced immunosuppressive properties to significantly reduce the symptoms of GvHD [61], indicating potential clinical application of INFγ primed MSCs. Nevertheless, expression variability among MSCs, which could lead to different expression levels of immunomodulatory genes among the licensed MSCs, implied their different immunomodulatory potency after priming. Therefore, conditions for MSC priming, such as optimum priming time, the concentration of INFγ, and whether different MSCs could be adjusted to similar immunomodulatory potency by priming design, need to be refined. And experimental immunosuppression and immunomodulation strategies could also be applied to enhance the predictive value of preclinical studies with MSCs [87].

In essential, genetic and epigenetic variations contribute to functional variability among MSCs. Identification of functional markers of potency in unlicensed MSCs could facilitate our understanding of MSCs’ mechanisms of action and development of release potency assays for them as potency release criterion [58]. IFNγ stimulation of MSCs recapitulates the molecular genetic changes that are observed in MSCs co-cultured with activated PBMCs [19]; however, it is necessary to notice that therapeutic effects of MSCs are multifaceted synergistic responses, which could form a balanced inflammatory and regenerative micro-environment in the presence of vigorous inflammation [1], and assays just focused on one functional aspect of MSCs, such as immunosuppressive potency, may ignore other functional capabilities of MSCs, which may also in some extend link to clinical results. Our analysis demonstrated that transcriptome-wide sample-to-sample variations among MSCs are associated with various functional properties (Fig. 2). Besides, their functional similarity and disparity can be classified based on expression of HVGs (Fig. 3), and thus, we speculated that these HVGs are valuable to serve as candidate matrix assays for potency analysis of MSCs without licensing. Comparison of response patterns to IFNγ among MSCs further showed that genes shared between CRGs and DRGs are significantly more variable than the other two sets (Fig. 6). Based on these genes’ expression, we established a primary model, which faithfully assessed immunosuppressive potency of unlicensed MSCs (Fig. 6). Beyond this, we inferred that RNA-seq technology combined with our model method can be extended to other functional variations of MSCs, such as interaction with innate immune cells and differentiation propensity.

## Conclusions

In summary, our study demonstrated that MSC samples can be classified into groups exhibiting distinct functional properties, such as immune modulatory potency, according to the expression pattern of HVGs. We also highlighted that MSCs deployed common regulation networks to exert immunosuppressive function while expression variability of genes in the networks could result in distinct immunosuppressive potency in MSCs. Finally, we found these different responsive genes showed high expression variability among unlicensed MSC samples as well, from which candidate markers were refined for development of matrix assays to quantify the immunosuppression potency of human unlicensed MSCs. In the future, with increased number of MSC samples, our analysis approach can be extended beyond immune modulatory potency to characterize other functional variations and related genes.

## Supplementary Information

**Additional file 1: Fig. S1.** Workflow for data search and selection. **Fig. S2.** Overview of data collected for transcriptome variation analysis. (**A**) Boxplot showing correlation coefficient of transcriptome expression for each sample to others in the dataset before data selection step 3. 0.9 was selected as a cutoff for filtering out samples with lower median Pearson’s r.(**B**) Barplot showing number of samples in each study. (**C**) Histograms showing number of reads (up) and mapping results (down) across samples. Some MSCs derived from different anatomical parts of placenta, AM CM, CP, and CV. AD: Adipose tissue; AM: Amniotic membrane; BM: Bone marrow; CM: Chorionic membrane; CP: Chorionic plate; CV: Chorionic villi; ED: Endometrial; DP: Dental pulp; PL: Placenta; UC: Umbilical cord. **Fig. S3.** Transcriptome variation across MSC samples**.** (**A**) Scatter plot showing DM value and mean expression (CPM) for each expressed gene. (**B**) Scatter plot showing DM value and gene length for each expressed gene. (**C**) GESA positive results showing enrichment in GO cellular component (left) and molecular function (right) gene sets database based on ranked genes list in descending order by the DM value. (**D**) GESA negative results showing enrichment in GO gene sets database based on ranked genes list in descending order by the DM value. Only the top 20 terms with highest NES were presented (*p* < 0.001). **Fig. S4.** Differential gene expression and function enrichment analysis of MSCs among G0, G2, G3 and G4. (**A**) Results of GO biological process enrichment analysis for genes upregulated in G0. (**B**) Representative genes upregulated in G0. (**C**) Results of GO biological process enrichment analysis for genes upregulated in G2. (**D**) Representative genes upregulated in G2. (**E**) Results of GO biological process enrichment analysis for genes upregulated in G3. (**F**) Representative genes upregulated in G3. (**G**) Results of GO biological process enrichment analysis for genes upregulated in G4. (H) Representative genes upregulated in G4. **Fig. S5.** Expression variability of MSCs in response to IFNγ. (**A**) Barplot showing genes responded differently to the IFNγ among ssG0, ssG2, and ssG4. (**B**) Venn diagraming showing overlap of genes responded differently to the IFNγ among ssG0, ssG2, and ssG4. (**C-E**) Barplot showing enrichment of genes responded differently to the IFNγ for ssG0 (C), ssG2(D), and ssG4 (E). **Fig. S6.** PCA visualizing distance of samples based on different gene panels. (**A**) Scatter plot visualizing PCA results using all genes in Genes set1 expressed in the untreated samples. (**B**) Scatter plot visualizing PCA results using all genes in Genes set2 expressed in the untreated samples. (**C**) Scatter plot visualizing PCA results using all genes in Genes set3 expressed in the untreated samples. (**D**) Scatter plot visualizing PCA results using HVGs expressed in the untreated samples. (**E**) Scatter plot visualizing PCA results using random sampling 100 genes expressed in the untreated samples. (**F**) Scatter plot visualizing PCA results using top 100 genes with the highest DM values in the Genes set2 expressed in the untreated samples and untreated samples.

**Additional file 2: Table S1.** Table S1 Information of MSCs RNA-seq samples downloaded from public GEO. **Table S2**. DM values for each gene expressed in MSC samples. **Table S3**. Information of samples clustered into different groups. **Table S4** Results of different expression analysis among G0, G2, G3, and G4 groups. **Table S5** Results of different expression analysis for INFγ treated vs untreat MSC paried samples. **Table S6.** List of genes differently respond to IFNγ treatment among ssG0, ssG2, and ssG4**. Table S7.** Overlap of genes between DRGs and CRGs.

## Data Availability

The data that support the findings of this study have been deposited in the CNSA (https://db.cngb.org/cnsa/) of CNGBdb with accession number CNP0000966. All data analyzed during this study have been included in this published article and its supplementary information files.

## Abbreviations

AD: Adipose tissue
BM: Bone marrow
DCs: Dendritic cells
DEGs: Differentially expressed genes
DM: Distance-to-median
DP: Dental pulp
DRGs: Different response genes
ED: Endometrial
GSEA: Gene set enrichment analysis
GvHD: Graft-versus-host disease
INFγ: Interferon gamma
ISCT: International Society for Cellular Therapy
MSCs: Mesenchymal stromal cells
NO: Nitrogen oxide
NOG: Noggin
NT5E: 5′-Nucleotidase
PBMCs: Peripheral blood mononuclear cells
TPM: Transcripts per kilobase million
tSNE: t-distributed stochastic neighbor embedding
UC: Umbilical cord
