# Supplementary material for: Deconstructing transcriptional variations and their effects on immunomodulatory function among human mesenchymal stromal cells
Source: Stem Cell Res Ther. 2021 Jan 9;12:53. doi: 10.1186/s13287-020-02121-8 (PMC7796611; doi:10.1186/s13287-020-02121-8)
Supplement: Supplementary file 1 — Additional file 1: Fig. S1. Workflow for data search and selection. Fig. S2. Overview of data collected for transcriptome variation analysis. (A) Boxplot showing correlation coefficient of transcriptome expression for each sample to others in the dataset before data selection step 3. 0.9 was selected as a cutoff for filtering out samples with lower median Pearson’s r.(B) Barplot showing number of samples in each study. (C) Histograms showing number of reads (up) and mapping results (down) across samples. Some MSCs derived from different anatomical parts of placenta, AM CM, CP, and CV. AD: Adipose tissue; AM: Amniotic membrane; BM: Bone marrow; CM: Chorionic membrane; CP: Chorionic plate; CV: Chorionic villi; ED: Endometrial; DP: Dental pulp; PL: Placenta; UC: Umbilical cord. Fig. S3. Transcriptome variation across MSC samples. (A) Scatter plot showing DM value and mean expression (CPM) for each expressed gene. (B) Scatter plot showing DM value and gene length for each expressed gene. (C) GESA positive results showing enrichment in GO cellular component (left) and molecular function (right) gene sets database based on ranked genes list in descending order by the DM value. (D) GESA negative results showing enrichment in GO gene sets database based on ranked genes list in descending order by the DM value. Only the top 20 terms with highest NES were presented (p < 0.001). Fig. S4. Differential gene expression and function enrichment analysis of MSCs among G0, G2, G3 and G4. (A) Results of GO biological process enrichment analysis for genes upregulated in G0. (B) Representative genes upregulated in G0. (C) Results of GO biological process enrichment analysis for genes upregulated in G2. (D) Representative genes upregulated in G2. (E) Results of GO biological process enrichment analysis for genes upregulated in G3. (F) Representative genes upregulated in G3. (G) Results of GO biological process enrichment analysis for genes upregulated in G4. (H) Representative genes upre [file 13287_2020_2121_MOESM1_ESM.docx]

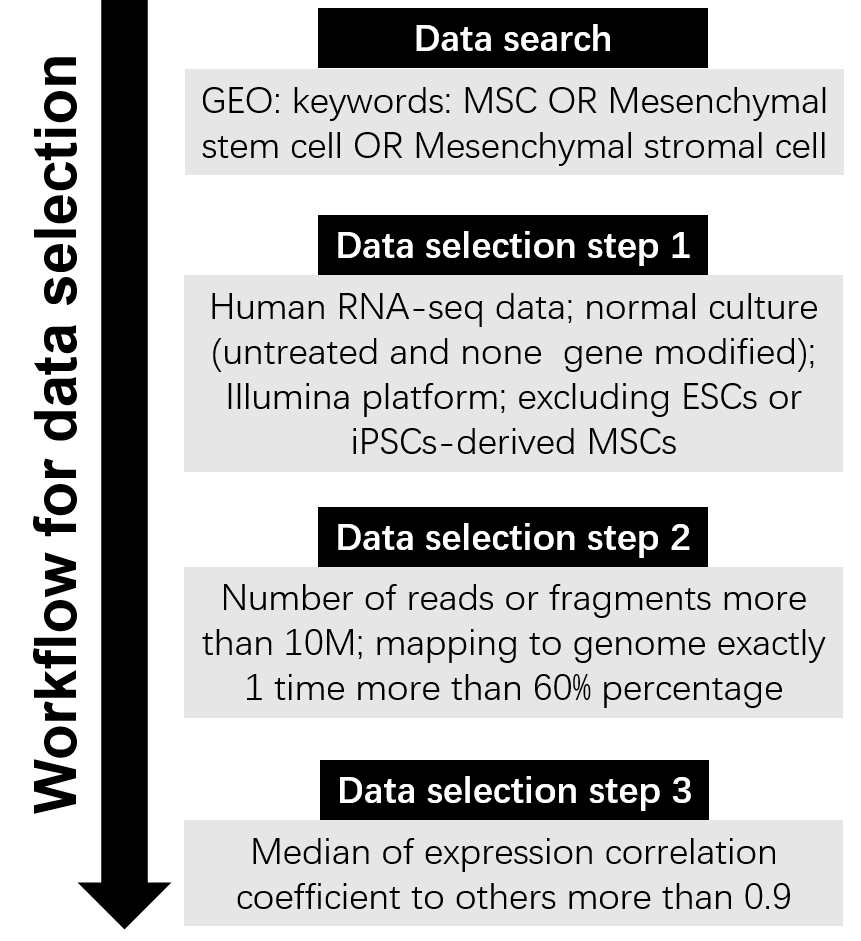


**Figure S1.** Workflow for data search and selection.


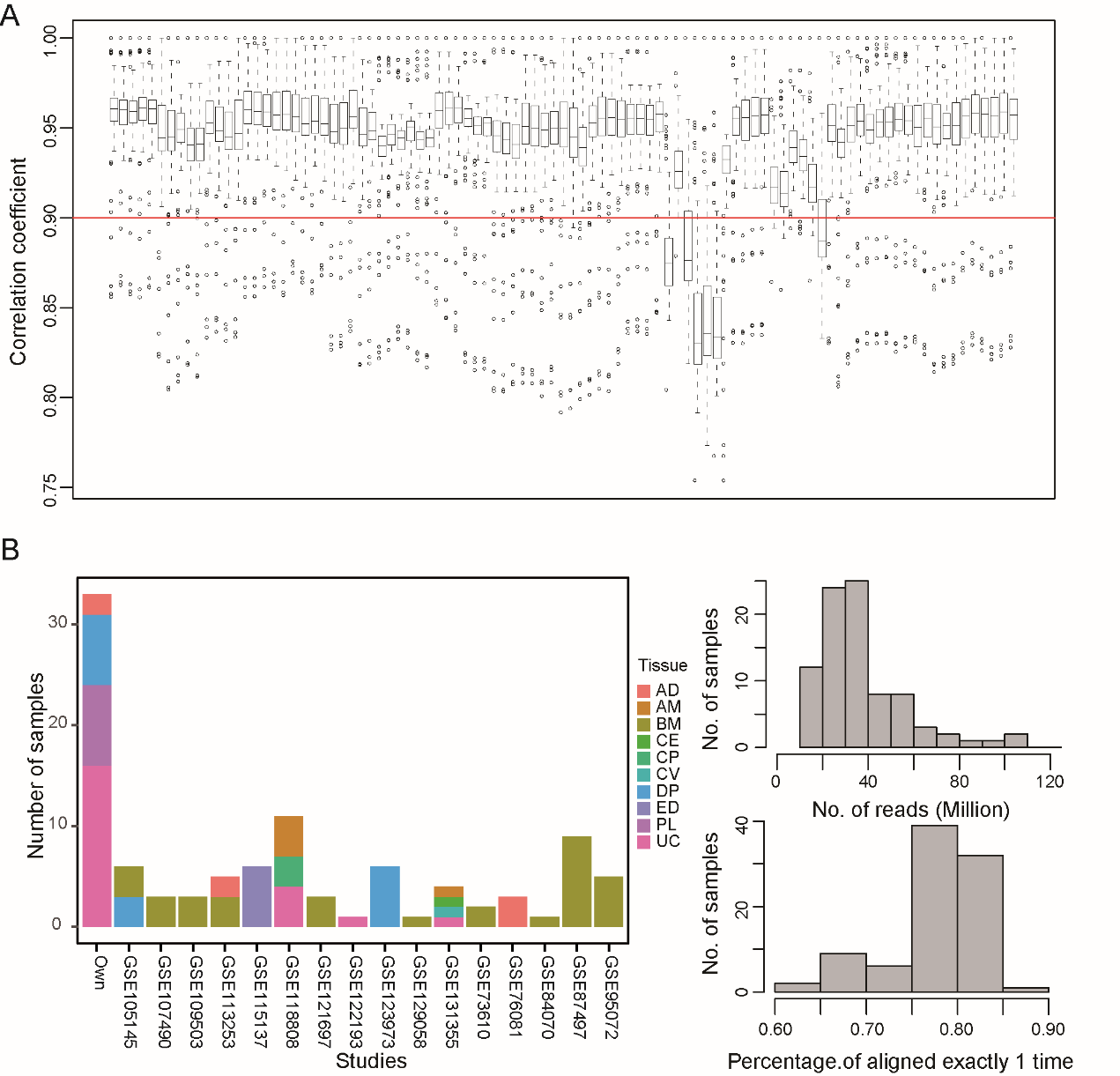


**Figure S2.** Overview of data collected for transcriptome variation analysis. (**A**) Boxplot showing correlation coefficient of transcriptome expression for each sample to others in the dataset before data selection step 3. 0.9 was selected as a cutoff for filtering out samples with lower median Pearson's r.(**B**) Barplot showing number of samples in each study. (**C**) Histograms showing number of reads (up) and mapping results (down) across samples. Some MSCs derived from different anatomical parts of placenta, AM CM, CP, and CV. AD: Adipose tissue; AM: Amniotic membrane; BM: Bone marrow; CM: Chorionic membrane; CP: Chorionic plate; CV: Chorionic villi; ED: Endometrial; DP: Dental pulp; PL: Placenta; UC: Umbilical cord.


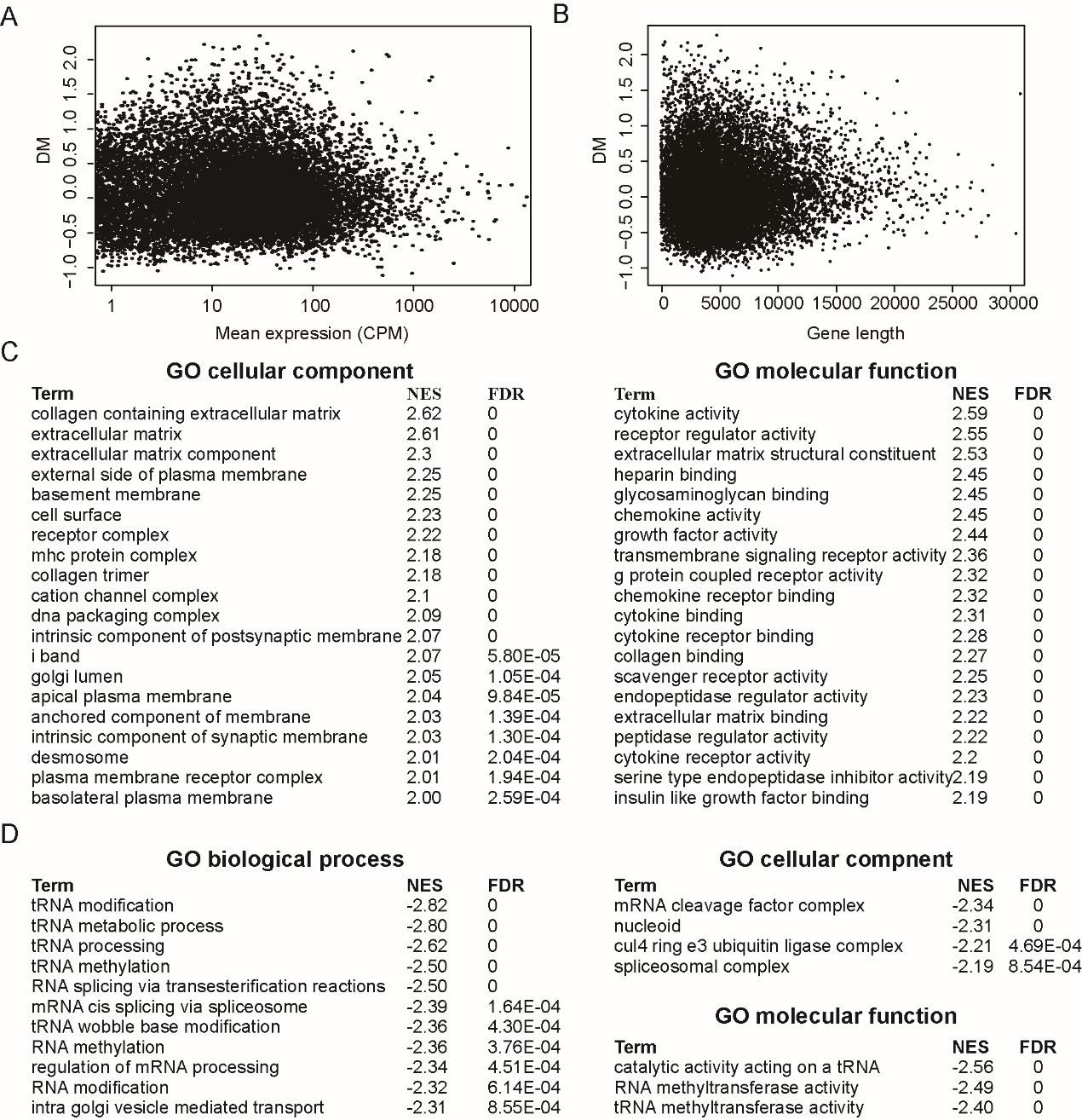


**Figure S3.** Transcriptome variation across MSC samples**.** (**A**) Scatter plot showing DM value and mean expression (CPM) for each expressed gene. (**B**) Scatter plot showing DM value and gene length for each expressed gene. (**C**) GESA positive results showing enrichment in GO cellular component (left) and molecular function (right) gene sets database based on ranked genes list in descending order by the DM value. (**D**) GESA negative results showing enrichment in GO gene sets database based on ranked genes list in descending order by the DM value. Only the top20 terms with highest NES were presented (p<0.001).


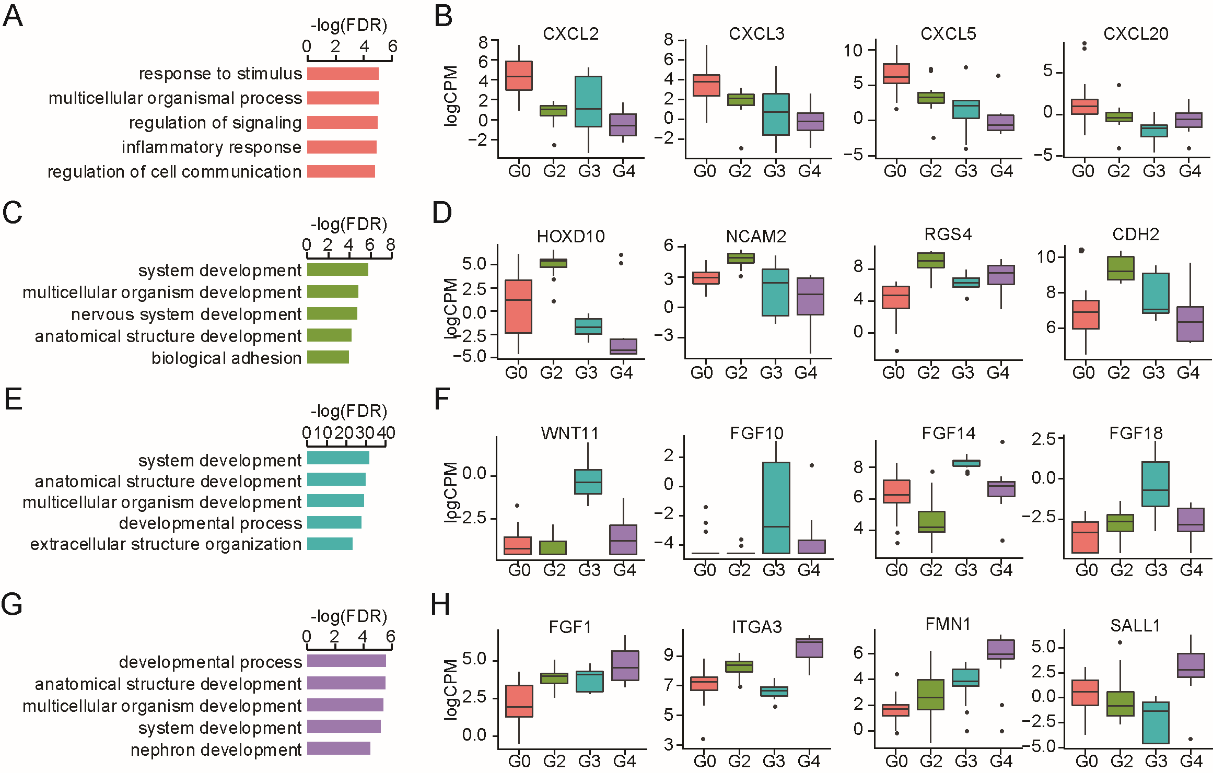


**Figure S4.** Differential gene expression and function enrichment analysis of MSCs among G0, G2, G3 and G4. (**A**) Results of GO biological process enrichment analysis for genes upregulated in G0. (**B**) Representative genes upregulated in G0. (**C**) Results of GO biological process enrichment analysis for genes upregulated in G2. (**D**) Representative genes upregulated in G2. (**E**) Results of GO biological process enrichment analysis for genes upregulated in G3. (**F**) Representative genes upregulated in G3. (**G**) Results of GO biological process enrichment analysis for genes upregulated in G4. (H) Representative genes upregulated in G4.


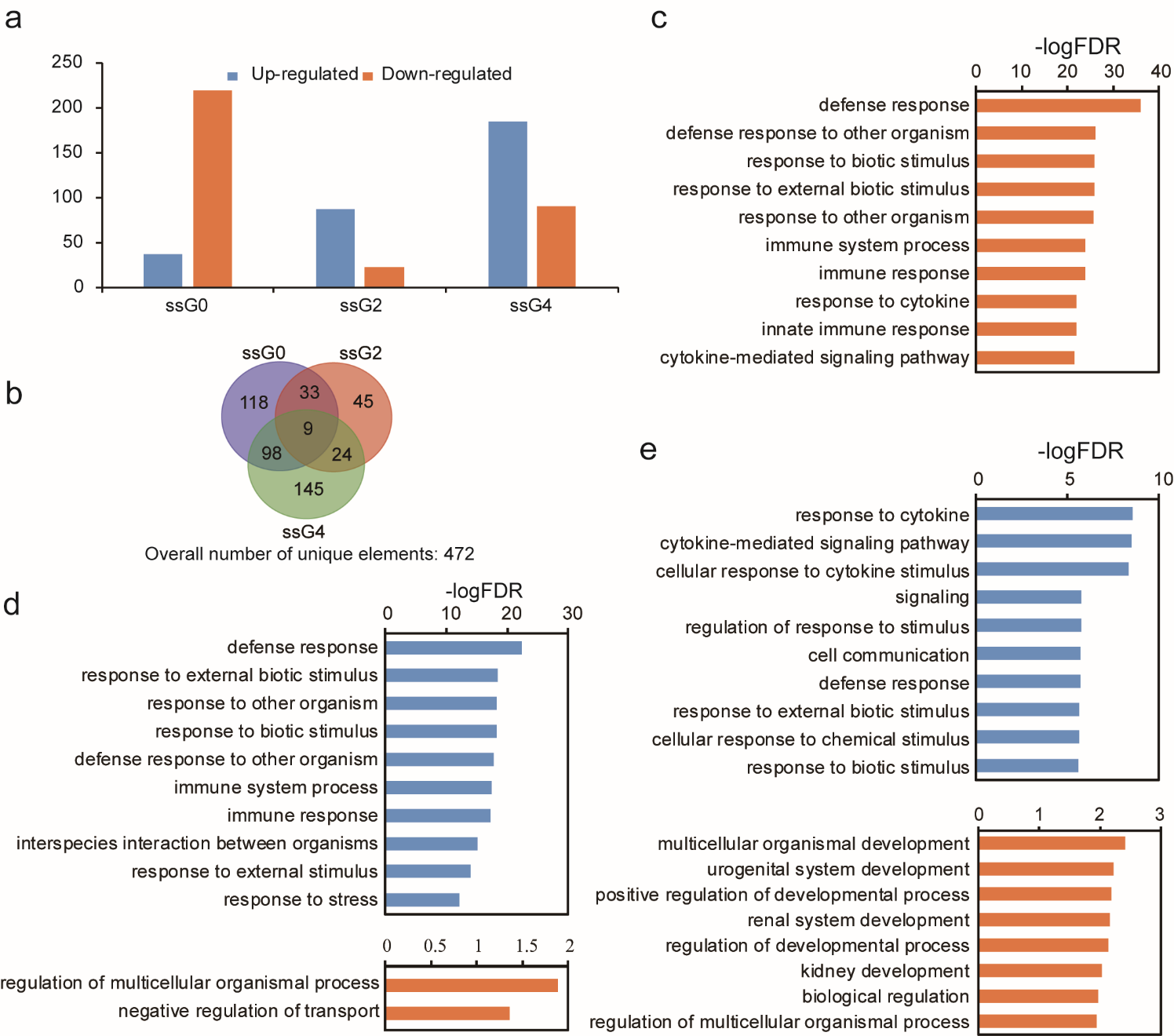


**Figure S5.** Expression variability of MSCs in response to IFNγ. (**A**) Barplot showing genes responded differently to the IFNγ among ssG0, ssG2, and ssG4. (**B**) Venn diagraming showing overlap of genes responded differently to the IFNγ among ssG0, ssG2, and ssG4. (**C-E**) Barplot showing enrichment of genes responded differently to the IFNγ for ssG0 (C), ssG2(D), and ssG4 (E).

**
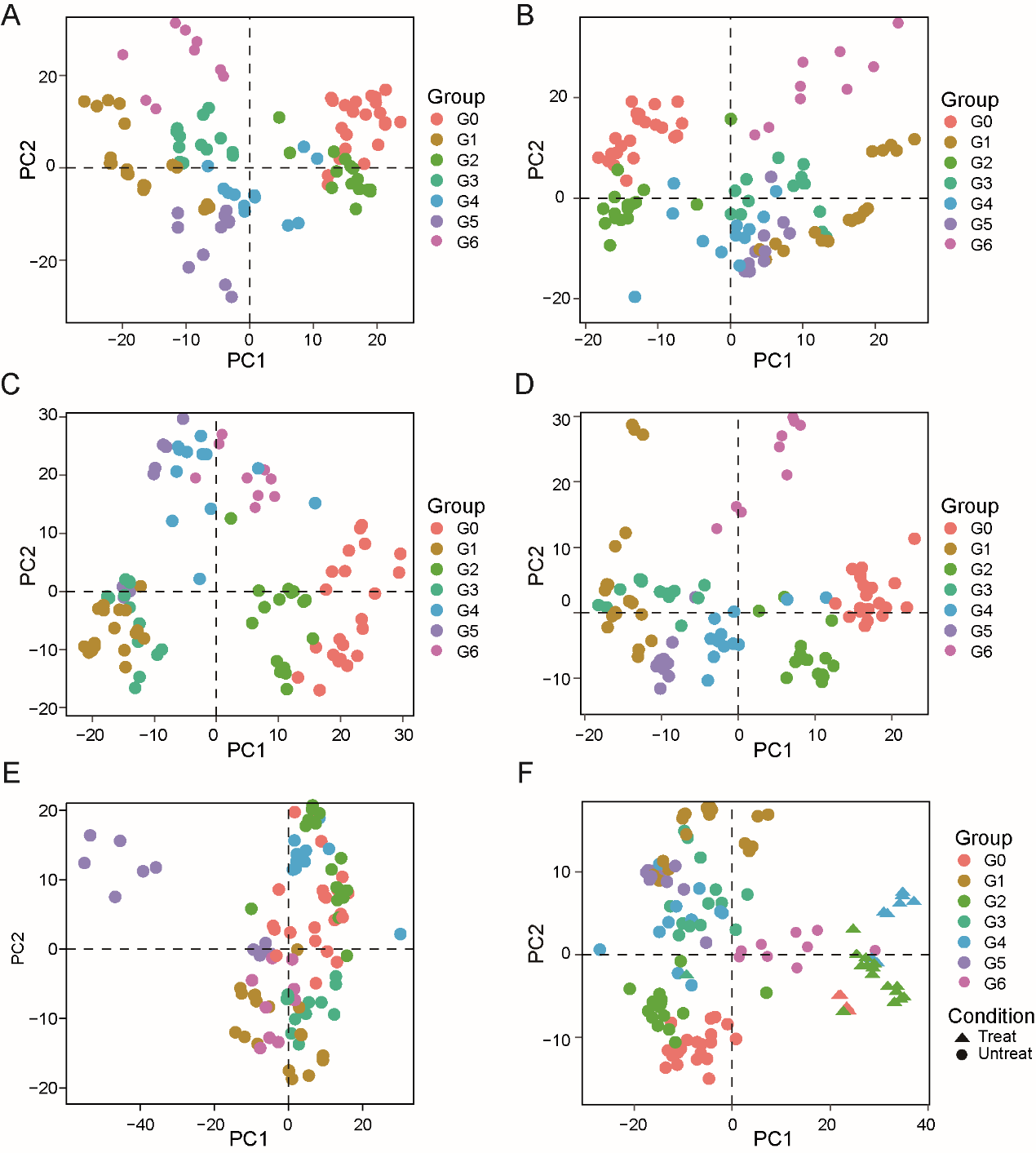
**

**Figure S6.** PCA visualizing distance of samples based on different gene panels. (**A**) Scatter plot visualizing PCA results using all genes in Genes set1 expressed in the untreated samples. (**B**) Scatter plot visualizing PCA results using all genes in Genes set2 expressed in the untreated samples. (**C**) Scatter plot visualizing PCA results using all genes in Genes set3 expressed in the untreated samples. (**D**) Scatter plot visualizing PCA results using HVGs expressed in the untreated samples. (**E**) Scatter plot visualizing PCA results using random sampling 100 genes expressed in the untreated samples. (**F**) Scatter plot visualizing PCA results using top100 genes with the highest DM values in the Genes set2 expressed in the untreated samples and untreated samples.
